# U-IMPACT: a universal 3D microfluidic cell culture platform

**DOI:** 10.1038/s41378-022-00431-w

**Published:** 2022-12-05

**Authors:** Seung-Ryeol Lee, Youngtaek Kim, Suryong Kim, Jiho Kim, Seonghyuk Park, Stephen Rhee, Dohyun Park, Byungjun Lee, Kyusuk Baek, Ho-Young Kim, Noo Li Jeon

**Affiliations:** 1 Department of Mechanical Engineering, Seoul National University, Seoul, Republic of Korea; 2 Qureator Incorporation, San Diego, CA USA; 3 Institute of Advanced Machines and Design Seoul National University, Seoul, Republic of Korea; 4 Institute of Bioengineering, Seoul National University, Seoul, Republic of Korea

**Keywords:** Microfluidics, Nanofabrication and nanopatterning

## Abstract

The development of organs-on-a-chip has resulted in advances in the reconstruction of 3D cellular microenvironments. However, there remain limitations regarding applicability and manufacturability. Here, we present an injection-molded plastic array 3D universal culture platform (U-IMPACT) for various biological applications in a single platform, such as cocultures of various cell types, and spheroids (e.g., tumor spheroids, neurospheres) and tissues (e.g., microvessels). The U-IMPACT consists of three channels and a spheroid zone with a 96-well plate form factor. Specifically, organoids or spheroids (~500 μm) can be located in designated areas, while cell suspensions or cell-laden hydrogels can be selectively placed in three channels. For stable multichannel patterning, we developed a new patterning method based on capillary action, utilizing capillary channels and the native contact angle of the materials without any modification. We derived the optimal material hydrophilicity (contact angle of the body, 45–90°; substrate, <30°) for robust patterning through experiments and theoretical calculations. We demonstrated that the U-IMPACT can implement 3D tumor microenvironments for angiogenesis, vascularization, and tumor cell migration. Furthermore, we cultured neurospheres from induced neural stem cells. The U-IMPACT can serve as a multifunctional organ-on-a-chip platform for high-content and high-throughput screening.

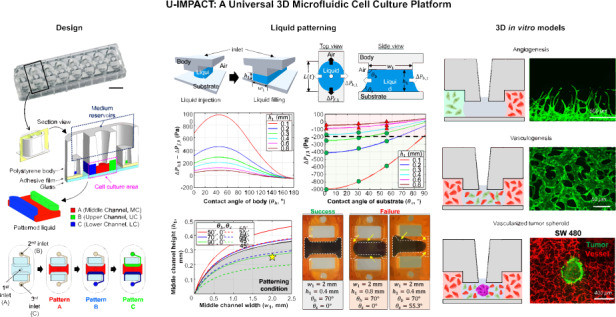

## Introduction

Organ-on-a-chip platforms recapitulate the physiological and pathological conditions in vivo (e.g., lung, brain, and liver) to gain a better understanding of disease mechanisms and allow more efficient drug development^1–3^. There is considerable interest in the possibility of using organ-on-a-chip platforms to replace or complement current in vitro and in vivo models^4,5^. Therefore, multiple studies have been performed to develop more reliable and reproducible platforms while more precisely mimicking aspects of the human body.

The tumor microenvironment (TME) is a very complex cellular and molecular microenvironment that consists of tumor tissue, blood vessels, fibroblasts, immune cells, and extracellular matrix (ECM)^6–8^. Although many polydimethylsiloxane (PDMS)-based platforms have been developed^9–11^, the applications to high-throughput screening (HTS) and high-content screening (HCS) remain limited^12^. These platforms have not followed the Society for Laboratory Automation and Screening [formerly Society for Biomolecular Screening (SBS)] format, resulting in poor compatibility with multichannel pipettes and HCS equipment. Furthermore, small molecule absorption and limited mass production capability hinder their practical use in the pharmaceutical industry and in hospitals^13,14^.

To develop tumor-on-a-chip platforms for HTS and HCS, a standardized organ-on-a-chip has recently emerged, consisting of a 96-well plate format microfluidic platform compatible with conventional equipment^15–17^. These microfluidic platforms represent new approaches to materials, fabrication processes, and patterning methods^18,19^. Some studies utilized lithography or milling processes and designed simple microstructures to pattern hydrogels and cells^19–21^. MIMETAS shows a well-formatted platform fabricated with photolithography on glass using a photoresist, which guides liquid behavior. AIM Biotech offers a plastic-based platform with a micropost array to maintain the integrity of hydrogels. Although they show various biological applications, including reconstruction of the TME, these microstructures lower the design flexibility, which limits scalability to another architecture. Other research groups introduced 3D printing or injection molding^10,22,23^. The liquid could be controlled by modifying the surface properties through plasma treatment or surface coating^15,19^. However, improvements to these platforms are still needed because of the design limitations in terms of manufacturability.

In this study, we developed an injection-molded plastic array 3D universal culture platform (U-IMPACT) that can be used for the TME and neurospheres. The U-IMPACT is a 96-well formatted microfluidic platform without microstructures and surface modifications. The platform consists of three channels and a spheroid zone for coculturing various cells and spheroids. For manufacturability and usability, we developed a hybrid liquid patterning method that integrates the principles of spontaneous capillary flow (SCF) and the capillary burst valve in our open microfluidic platform. After the establishment of design criteria, we fabricated the U-IMPACT by bonding a polystyrene body and glass with an adhesive film without additional processing, such as surface treatment or coating. We successfully recapitulated the microenvironment of angiogenesis, perfused blood vessels, tumor cell migration, and vascularized tumor spheroids in the U-IMPACT. Furthermore, we confirmed neural cell culture (e.g., differentiation of induced neural stem cells [iNSCs]) in our platform. We expect the U-IMPACT to be useful as a versatile microfluidic platform for the reconstruction of various human disease models, as well as applications to HCS or HTS.

## Results

### Design concept of the U-IMPACT

**Fig. 1:**
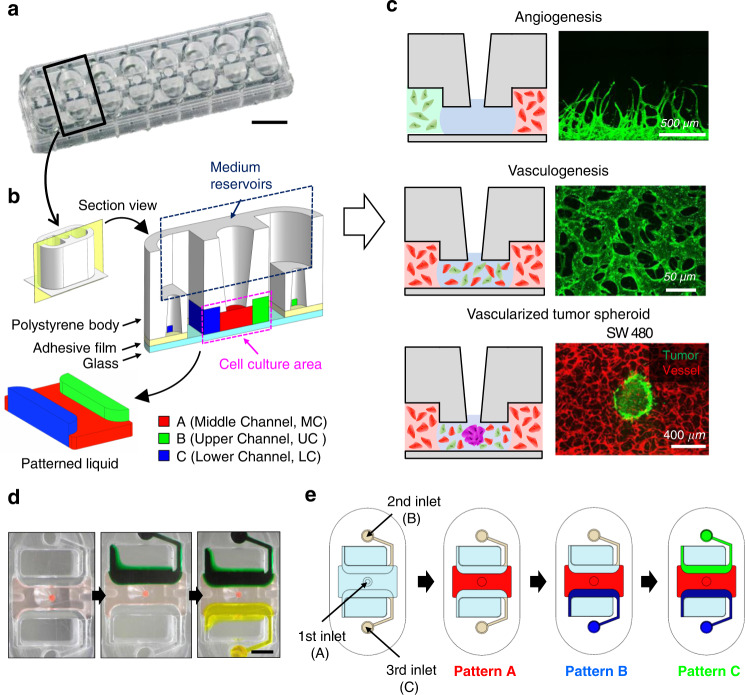
Overview of the U-IMPACT. **a** Photograph of the U-IMPACT. The platform has a 96-well plate format and slide glass size. Scale bar, 1 cm. **b** Schematic illustration of the U-IMPACT. The U-IMPACT is a standardized microfluidic platform with two combined 96-well plate formats. The section view shows two distinct medium chambers separated by a wall (navy dotted line) and three channels (red, green, and blue) for each inlet. Various cells or extracellular matrix can be introduced into these channels through the port. The MC (red) has a spheroid zone. The platform consists of the body (white), adhesive film (gray), and substrate (light blue). **c** Various biological applications are performed using the U-IMPACT platform. The applications include angiogenesis, perfusable vessel networks, and tumor-induced angio/vasculogenesis. **d** Patterning process and results. Scale bar, 2 mm. **e** Schematic illustration of the detailed patterning process. The MC is filled with hydrogel through the first inlet (red). After fibrin crosslinks and solidifies in the MC, the side channel is filled with hydrogel or liquid through the side inlet (blue or green, respectively).

We designed the U-IMPACT as a reproducible and reliable standardized microfluidic platform for HCS or HTS, focusing on fabrication and hydrogel patterning. The U-IMPACT consists of three parts: the body, double-sided adhesive film, and substrate (Fig. 1a). Because the adhesive film is easily attachable/detachable without any other processing (e.g., plasma treatment), we could simply conduct experiments with various body and substrate materials to identify suitable combinations for reliable patterning. Based on the results of theoretical analysis and patterning experiments, we settled on injection-molded polystyrene (PS) for the body with a glass substrate (Fig. 1b).

For compatibility with automation and imaging equipment, the U-IMPACT has a 96-well plate format with a slide glass size of 3 × 1 inch. In addition, for the coculture of various cells and tumor spheroid, we designed a platform with multiple channels and spheroid inlets. Specifically, the platform consists of three hydrogel channels (magenta dotted line, Fig. 1a), three inlets connected to each channel, and a wall separating the well into two medium reservoirs (navy dotted line, Fig. 1a). The middle inlet enables the injection of hydrogel or spheroids. The middle channel (MC, shown in red, Fig. 1a) has a lower height than the two side channels (upper channel, UC; lower channel, LC; shown in green and blue, respectively, Fig. 1a). This height difference allows selective liquid patterning, acting as a capillary burst valve. The patterning sequence is as follows: the MC is filled through the middle inlet, while the two side channels (UC and LC) are filled through each port (Fig. 1c, d). For cell culture experiments, the medium reservoirs are filled after hydrogel fibrillogenesis and cell suspension stabilization.

### Measurement of platform materials

Our goal was to develop the U-IMPACT as a ready-to-use microfluidic platform. Although patterning by SCF enables robust control of liquid, plasma treatment is required before experiments, which reduces manufacturability^15,20^. Therefore, we decided to utilize the natural properties of materials without any other processing, and we measured the static and advancing contact angles of various body and substrate materials (Fig. S1a, b). We investigated two materials for the body: polystyrene and 3D printer material (3DP). Polystyrene has been widely adopted as a material for Petri dishes, and 3DP is suitable for parameter analysis^10,18^. In addition, assuming that the hydrogel in the MC acts as a solid state during side channel patterning, the fibrin gel contact angle was measured. We also used five materials with different contact angles as the substrate: glass, 3M hydrophilic film, acrylic polyethylene terephthalate (PET) film, polycarbonate (PC) film, and plasma-treated PC film. These materials were tested to determine the contact angle most suitable for patterning, although 3M hydrophilic film and acrylic PET film are unsuitable for cell culture.

Cleaning was performed by washing with isopropyl alcohol (IPA) and deionized water, treatment with plasma, and heating at 60 °C in an oven to eliminate dust or oil on polystyrene and 3DP during injection molding and posttreatment processes. Hydrophobic recovery was examined in both polystyrene and 3DP (Fig. S1c, d). The contact angle of polystyrene reached 70° after 7 days and remained almost constant for more than 1 month^24^. This value was lower than the original contact angle of 96°. Therefore, we concluded that the contact angle recovered, but it was difficult to reach the original angle. The same phenomenon was observed for 3DP. Notably, the contact angles of polystyrene and 3DP after the cleaning process were almost identical (approximately 70°), which supported parameter analysis using a 3DP prototype before injection molding with polystyrene.

### Principle of patterning in the U-IMPACT

**Fig. 2:**
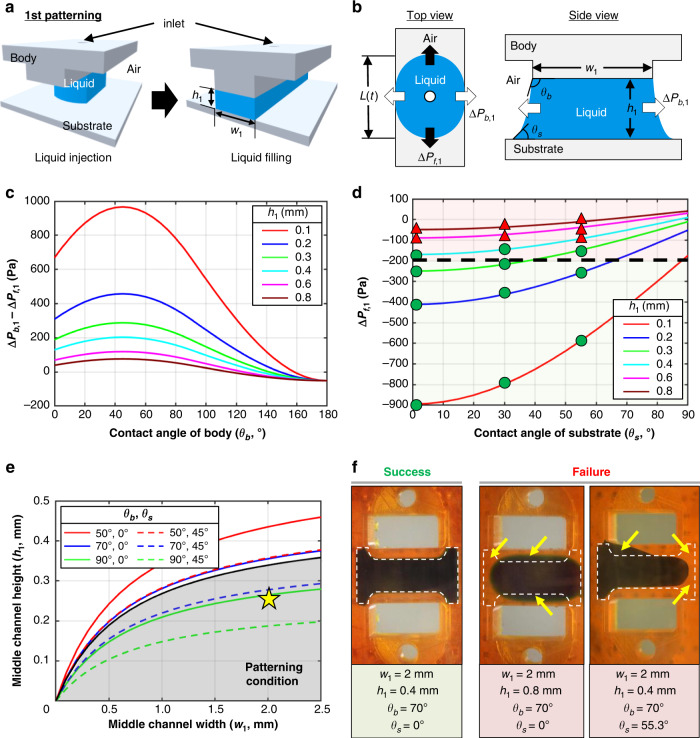
Liquid patterning on the MC. **a** Schematic illustration of filling the MC through the first inlet. Under specific conditions, liquid can be pinned on the body structure and proceed toward the end of the channel. **b** There are two major pressures at the liquid interface during the gel-filling process: capillary burst pressure (∆*P*_b*,*1_) and internal pressure at the liquid front (∆*P*_f,1_). **c** Graph of the pressure difference (∆*P*_b,1_−∆*P*_f,1_) with a width of 2 mm. The pressure difference must be maximized to maintain the interface in the desired channel without bursting into the side channels. **d** Graph of ∆*P*_f,1_ with a body contact angle of 70° and a width of 2 mm. The ∆*P*_f,1_ must be minimized for complete filling. After parameter analysis, we empirically set ∆*P*_f_ to −200 Pa for robust patterning (green circles, success; red triangles, failure). **e** Patterning conditions in the MC. We established design rules for contact angles and dimensions. The gray area and yellow star indicate the contact angles (body, 70°; substrate, 20°) and dimensions (width, 2 mm; height, 0.25 mm) of the U-IMPACT. **f** Experimental results of liquid patterning (green).

**Fig. 3:**
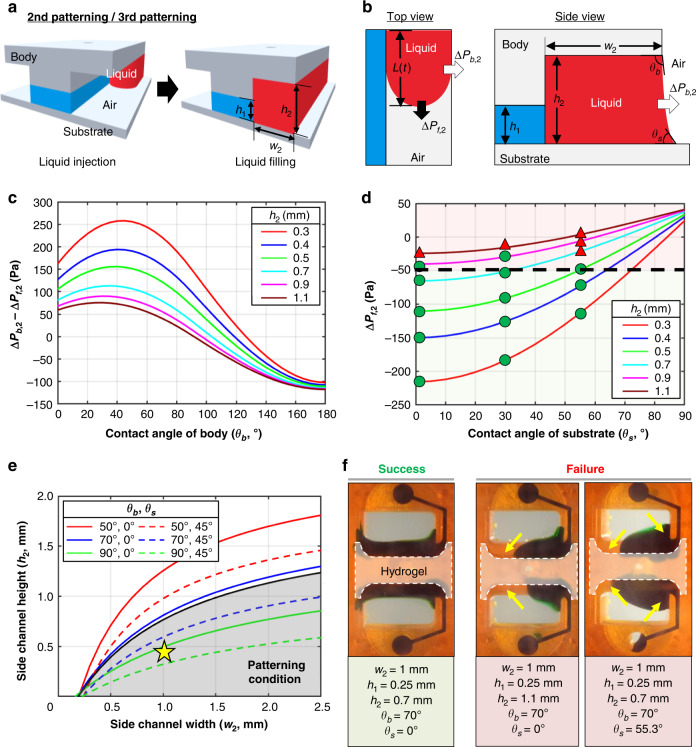
Liquid patterning on the side channel. **a** Schematic illustration of side channel filling through the side inlet. **b** Two major pressures (∆*P*_b,2_, ∆*P*_f,2_) at the liquid interface during the gel-filling process. **c** Graph of the pressure difference (∆*P*_b,2_−∆*P*_f,2_) with a width of 1 mm. **d** Graph of ∆P_f,2_ with a body contact angle of 70° and width of 1 mm. After parameter analysis, we empirically set ∆*P*_f,2_ to −50 Pa for robust patterning (green circles, success; red triangles, failure). **e** Patterning conditions in the side channel. The gray area and yellow star indicate the contact angles (body, 70°; substrate, 20°) and dimensions (width, 1 mm; height, 0.45 mm) of the U-IMPACT. **f** Experimental results of liquid patterning (green). After hydrogel fibrillogenesis in the MC (white), the liquid was patterned in the side channel.

We developed a new hybrid patterning method combining the principles of the capillary burst valve and SCF. We designed a height difference (instead of microposts) for the capillary burst valve and then utilized the natural properties of the materials (i.e., the contact angle) instead of plasma treatment for SCF. Two patterning cases were investigated: MC and side channel patterning (Figs. 2a and 3a). The first patterning involved filling the MC, while the second and third patterning filled both side channels (UC and LC). Because the UC and LC are symmetrical, we describe only the second patterning in the UC here.

All patterning cases were balanced by capillary forces and surface tension. During the gel-filling process, two major pressures were created at the interface of the liquid (Figs. 2b, 3b). One pressure [${\Delta}{{{P}}}_{{{{{\rm {forward}}}}}},({\Delta}{{{P}}}_{{{\rm {{f}}}}})$] was generated at the interface where the liquid advanced, while the other pressure [${\Delta}{{{P}}}_{{{{\rm {{burst}}}}}},\left( {{\Delta}{{{P}}}_{{{\rm {{b}}}}}} \right)$] was generated at the interface where the liquid was pinned. These pressures are determined by the geometries of the channel (*w*, width; *h*, height; *L*, length), advancing contact angle of the body (*θ*_b_) and substrate (*θ*_s_), and surface tension (*γ*). We set the maximum length to 7 mm, which is the diameter of the wells in the 96-well plate format. We assumed that the injection pressure, which is the external force applied to the flow liquid, was satisfied within the range between ∆*P*_f_ and ∆*P*_b_. The pressure difference (${\Delta}{{{P}}}_{{{\rm{b}}}} - {\Delta}{{{P}}}_{{{\rm{f}}}}$) must be maximized to maintain the integrity of the liquid interface between channels by means of the capillary burst valve, while ∆*P*_f_ must be minimized to allow complete filling of the desired channel by SCF^19,25^. Therefore, we defined successful hybrid patterning only when these two conditions were satisfied.

### Liquid patterning in the U-IMPACT

We analyzed the effects of contact angle and dimension on liquid patterning. For the first patterning, the liquid was injected through the first inlet and filled the MC (shown in blue, Fig. 2a). The MC is defined by two solid–liquid interfaces (ceiling and floor) and two air–liquid interfaces (open surface to UC and LC). Using the total interfacial energy of the system (see the “Methods” section), we evaluated the two pressures, ∆*P*_f,1_ and ∆*P*_b,1_ (Fig. 2b)^20^.

The pressure difference (∆*P*_b,1_ and ∆*P*_f,1_) is expressed as a function of the contact angle of the body and dimension (Figs. 2c, S2b). Because the pressure difference must be maximized to maintain the liquid interface by the capillary valve, a lower height and wider width are preferred. In addition, a hydrophilic body is more suitable for patterning than a hydrophobic body. However, for contact angles <45°, SCF occurs, and liquid flows into a corner or wedge in accordance with the Concuss–Finn relationship (Fig. S2a). Therefore, we concluded that a body contact angle in the range of 45–90° was suitable. Next, the pressure ∆*P*_f,1_ is expressed as a function of the dimension and contact angles of the body and substrate (Fig. 2d, S2c–e). Because ∆*P*_f,1_ must be minimized for complete filling of the desired channel by SCF, a substrate contact angle closer to 0° is preferred. In addition, similar to the pressure difference, a lower height and wider width are more suitable for patterning.

We performed experiments with varying heights and materials (green circles, success; red triangles, failure in Figs. 2d, S2d). Specifically, we conducted a parameter analysis with the body (3DP body with or without plasma treatment) and substrate (PC film with plasma treatment, 3M hydrophilic film, and acrylic PET film). The results indicated that the success rate of patterning was high when (1) the body contact angle was between 45° and 90°, (2) the substrate contact angle was close to 0°, and (3) the height was low. Based on the results of the experiments, we empirically determined an ∆*P*_f,1_ of −200 Pa as the maximum threshold for successful patterning. From the analytical model and experiment, we established a design rule for the first liquid patterning in terms of the dimension and contact angles of the body and substrate (Fig. 2e). If the dimension or substrate does not satisfy these conditions, the liquid cannot be pinned at the edge of the body or advanced toward the end of the channel, and patterning is expected to fail (Fig. 2f).

For analysis of the second patterning, the liquid was injected through the side inlet and filled the UC after hydrogel fibrillogenesis in the MC (shown in red, Fig. 3a). Assuming that the hydrogel in the MC acts as a solid state, the UC can be defined by three solid–liquid interfaces and one air–liquid interface (open surface to medium reservoir). Accordingly, we evaluated two pressures, ∆*P*_f,2_ and ∆*P*_b,2_ (Fig. 3b). The pressure difference (∆*P*_b,2_−∆*P*_f,2_) and ∆*P*_f,2_ showed tendencies similar to the results in the first patterning (Figs. 3c, d, S3a–d). Patterning improved with decreasing height and increasing width. In addition, the body contact angle was appropriately in the range of 45–90°, while the substrate contact angle was closer to 0°. After parameter analysis, we experimentally set ∆*P*_f,2_ to −50 Pa as the maximum threshold for patterning (Figs. 3d, S3c). Therefore, we established design rules for both the first and second liquid patterning (Fig. 3e). If the dimension or substrate exceeded these conditions, patterning failed (Fig. 3f).

In summary, we developed a new hybrid multichannel patterning system by integrating SCF and capillary burst valve principles. We utilized the channel height difference for the capillary valve and the material contact angle for SCF. Based on the analytical model and experiments, we established design criteria for the dimension and contact angle. Based on our results, we constructed a platform comprising a polystyrene body (70°) and glass substrate (20°) (black line and gray area in Fig. 2e, f). Specifically, the MC was designed with a width of 2 mm and height of 0.25 mm, while the side channels (UC and LC) were designed with a width of 1 mm and height of 0.45 mm (yellow star in Fig. 3e, f). The diameter of the middle inlet for the injection of cell spheroids was 0.6 mm, while the diameter of the side inlet was 1.3 mm. We finely tuned the volume to 6 μL for the first patterning; both side channels required 11 μL to fill the channel completely.

### The reconstruction of TME in the U-IMPACT

Using three channels and a spheroid zone in a single platform, we reconstructed various TME models, such as angiogenesis, vasculogenesis, vascularized tumor, and tumor migration. Because a stable concentration gradient is essential for TME reconstruction, we performed COMSOL simulation to determine whether the design of the U-IMPACT was suitable for cell culture. To determine the critical limit of the concentration gradient, we analyzed the PDMS-based platform previously developed by our group for angiogenesis and vasculogenesis^26,27^. The mean concentration gradient in which endothelial cells (ECs) were seeded (B-B′, Fig. S4a–c) in the PDMS-based platform was 0.078%/mm; therefore, we regarded this as the minimum value.

**Fig. 4:**
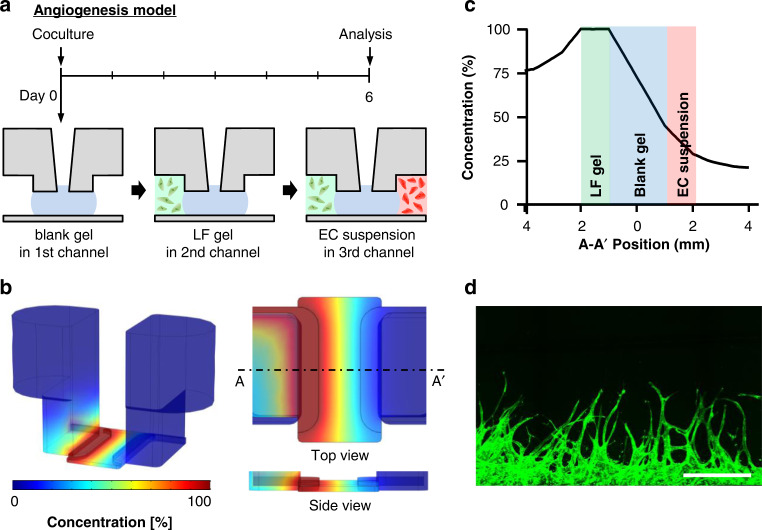
Modeling of 3D angiogenesis. **a** Timeline of the angiogenesis model. We injected hydrogel into the MC, introduced LFs with hydrogel into the LC, and added the EC suspension into the UC. **b** Simulation of growth factor concentration in the U-IMPACT. The growth factor source was the LC in which LFs or tumor cells were seeded. **c** Distribution of growth factor concentration in the middle of the U-IMPACT (A-A′). **d** Confocal imaging of ECs growing toward the LF channel. Scale bar, 500 μm.

**Fig. 5:**
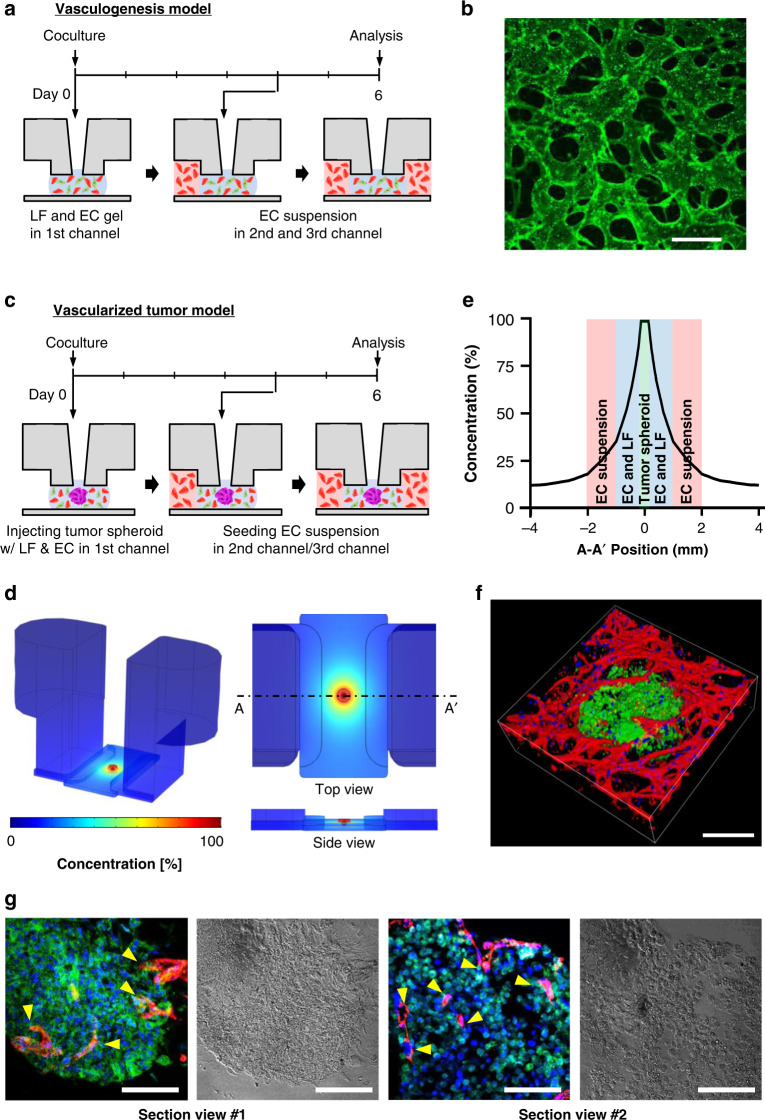
Modeling of vascularized tumor spheroid. **a** Timeline of the vasculogenesis model. The mixture of LFs, ECs, and hydrogel was injected into the MC. After 4 days, the EC suspension was added to both side channels. **b** Confocal imaging of the perfusable vascular network (green) on Day 6. Scale bar, 50 μm. **c** Timeline of the vascularized tumor model. The mixture of LFs, ECs, a tumor spheroid, and hydrogel was injected into the MC. After 4 days, the EC suspension was added to both side channels. **d** Simulation of growth factor concentration in the U-IMPACT. The tumor spheroid was the main source of growth factors. **e** Distribution of growth factor concentration in the middle of the U-IMPACT (A-A′). **f** Confocal imaging of vascularized tumor spheroid (green, U87MG; red, EC). Scale bar, 250 μm. **g** Imaging of sectioned tumor spheroid (left, immunofluorescence; right, bright field; blue, DAPI; green, U87MG; red, EC). Scale bar, 100 μm.

**Fig. 6:**
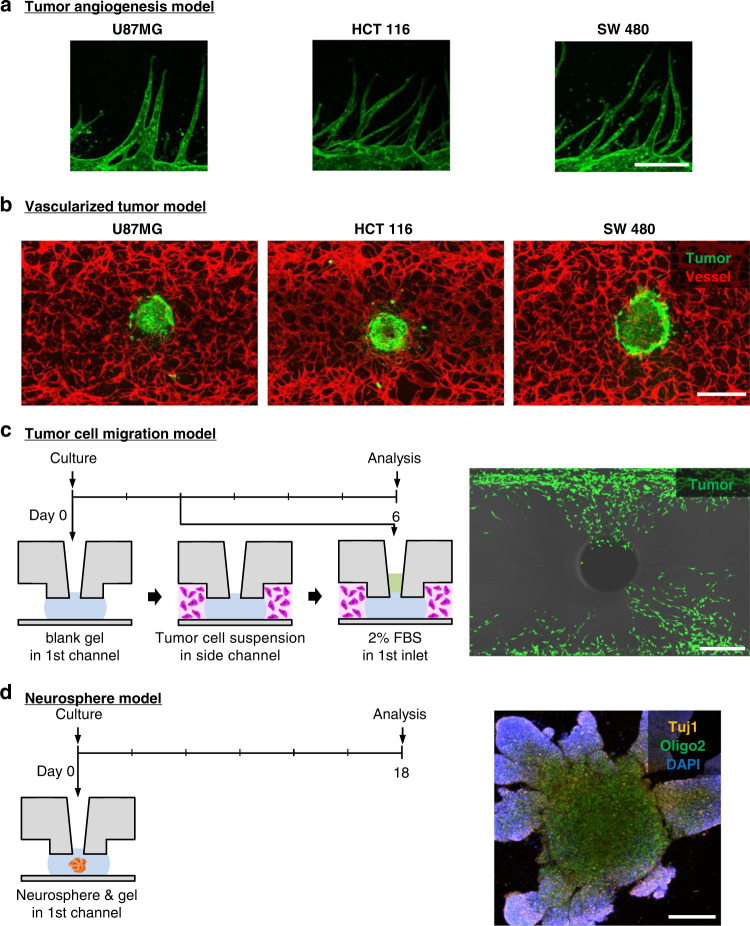
3D tumor and neural cell culture model in the U-IMPACT. **a** Confocal imaging of angiogenesis toward UC in which tumor cells and LF were seeded. We used three types of tumor spheroids composed of U87MG, HCT116, and SW 480 cells. Scale bar, 250 μm. **b** Confocal imaging of vascularized tumor spheroid. Scale bar, 400 μm. **c** Timeline (left) and confocal imaging (right) of tumor cell migration. We injected collagen type I gel into the MC and seeded the tumor cell suspension in the side channels. We then injected 2% FBS in the medium through the first inlet on Day 2. Tumor cells (green) migrated toward the first inlet. Scale bar, 500 μm. **d** Timeline (left) and confocal imaging (right) of neurosphere with iNSCs. We injected the neurosphere and hydrogel (Matrigel) into the MC. The iNSCs differentiated into neurons and oligodendrocytes (Tuj1, orange; Oligo2, green; DAPI, blue). Scale bar, 250 μm.

In the angiogenesis model, fibrin gel was injected into the MC (Fig. 4a). After the mixture of lung fibroblasts (LFs) and fibrin gel was injected into the LC, the EC suspension was injected into the UC. The LC became the source of growth factors because LFs released vascular endothelial growth factor and induced ECs to sprout toward areas of higher vascular endothelial growth factor concentrations^9^. The 3D simulation results revealed that a mean concentration of 40% (Fig. 4b, c) and concentration gradient of 0.17%/mm (Fig. S4d, e) were generated in the EC suspension channel after 12 h. Compared with the PDMS-based platform, the concentration gradient was sufficient to generate angiogenesis. After 6 days, we observed that ECs grew toward the LC in which LFs had been seeded (Fig. 4d). Furthermore, upon seeding of three cancer cell lines (i.e., U87MG, HCT116, and SW 480) with LFs in the LC, we confirmed that cancer cells also induced angiogenesis (Fig. 6a). In the vasculogenesis model, a mixture of fibrin gel, LFs, and ECs was injected into the MC (Fig. 5a). After 4 days, the EC suspension was seeded into both LC and UC. We confirmed the formation of a perfusable vascular network by a microbead assay (Fig. 5b, Movie S1). The 2 μm microbeads were perfused only through the lumen of an approximately 30 µm vascular network.

After demonstrating that the design of the U-IMPACT allows the formation of a 3D vascular network, we conducted vascularized tumor modeling experiments. After the injection of fibrin gel with LFs, ECs, and a tumor spheroid into the MC, the spheroid was placed in the middle of the MC because of the confined geometry (Fig. 5c). After 4 days, the EC suspension was attached to both the LC and UC. The simulation results showed that a mean concentration of 35% (Fig. 5d, e) and concentration gradient of 0.12%/mm (Fig. S4f, g) were generated in the EC suspension channel after 12 h. We confirmed various vascularized brain and colon cancer tumor models by confocal imaging (Figs. 5f, 6b). Additionally, we performed immunofluorescence imaging of the sectioned tumor spheroid to show vascularization in the platform (Fig. 5g). We also investigated tumor cell migration (Fig. 6c). After the injection of blank fibrin gel into the MC, the tumor cell suspension was injected into the side channel. After 2 days, 2% fetal bovine serum (FBS) in the medium was introduced into the first inlet. At 6 days, we found that tumor cells had migrated toward the first inlet.

## Discussion

Here, we present the U-IMPACT, a 3D versatile standardized microfluidic platform for the recapitulation of various organs, as well as applications in HTS and HCS. We optimized the design for manufacturability by fabricating the U-IMPACT with a polystyrene compartment, adhesive film, and glass substrate without additional processing (i.e., surface treatment). Furthermore, we developed a hybrid patterning method by integrating SCF and capillary burst valve principles and then established design criteria to pattern three channels and one spheroid zone. After demonstrating a concentration gradient by finite element methods, we reconstructed aspects of the 3D TME, such as angiogenesis, tumor migration, and vascularized tumors. We believe that our platform is a reliable and reproducible platform, enabling not only the reconstruction of diverse cellular and molecular microenvironments but also HTS and HCS.

Multiple organ-on-a-chip platforms have recently been developed for practical use in hospitals and the pharmaceutical industry^17,28^. Although many laboratories have developed novel in vitro platforms, improvements in reliability, reproducibility and usability remain necessary^12^. Considering these issues, we designed the U-IMPACT as follows. First, it is a mass-produced standardized platform (96-well plate format) that is compatible with existing equipment (e.g., multichannel pipettes and high-throughput imaging). Second, it comprises a ready-to-use microfluidic platform that will allow simple fabrication and robust patterning with spheroids, regardless of user proficiency. Third, it constitutes a 3D in vitro culture platform that will enable a stable growth factor concentration gradient to recapitulate the TME. The U-IMPACT allows reliable and reproducible fabrication and patterning, lowering the barrier to the access and use of organ-on-a-chip technology.

For simple fabrication and robust patterning, we developed a new hybrid patterning method facilitated by natural material properties and height differences. Our new approach to patterning has several advantages compared with conventional methods, such as SCF by plasma treatment in the hydrophilic state or a micropost array in the hydrophobic state^19,25,29,30^. First, our design has improved manufacturability, and the total fabrication time is reduced due to the omission of the plasma treatment step. Second, in terms of the shelf life of the product, it is no longer necessary to sustain a hydrophilic state by vacuum packing. Because hydrophobic recovery is a natural phenomenon, it is difficult to maintain hydrophilic conditions^24,31^. Third, design freedom is enhanced by eliminating the micropost array, leading to various new designs for injection-molded platforms. Combining analytical and experimental results, we established the dimension rules in terms of contact angle and dimensions. Consequently, U-IMPACT is a ready-to-use platform in industrial fields.

Importantly, our platform allows various coculture models using the combination of the three channels. This represents an advance over our previous tumor spheroid-on-a-chip model, the Sphero-IMPACT, which had only one channel; the U-IMPACT has three channels to allow diverse patterning of different types of cells in the desired channels^10^. In addition, simulation analysis demonstrated that the design of the U-IMPACT allows the formation of a stable concentration gradient for 3D coculture. Because our model showed the potential for reconstructing vascularized tumor spheroids with different types of gel or cell suspension, it will be of interest for analyzing vascular physiology or TME to establish how spheroid size affects the vascular network, which types of fibroblasts (e.g., cancer-associated fibroblasts) contribute to angiogenesis, and which factors drive angiogenesis in the stable 3D coculture microenvironment. In addition, we demonstrated neural cell culture models (neurospheres from iNSCs). Confocal imaging analysis confirmed that neurospheres differentiated into neurons and oligodendrocytes (Fig. 6d).

## Conclusion

We developed a versatile standardized 3D microfluidic culture platform, designated U-IMPACT, with three channels and one spheroid zone for various microenvironments. Designed for manufacturability, the U-IMPACT enables simple and robust patterning without any surface treatment or other additional processing by bonding polystyrene and glass with adhesive film. In addition, the U-IMPACT facilitates a new hybrid patterning method combining the SCF and capillary burst valve principles. We established design rules for the contact angle and dimensions. We recapitulated elements of the TME, such as angiogenesis, vasculogenesis, and a vascularized tumor spheroid. Furthermore, we cultured a neural spheroid from induced neural stem cells (iNSCs). The U-IMPACT is a versatile microfluidic platform for investigating TME and other human tissues.

## Experimental section

### Prototype of the U-IMPACT

A prototype of the U-IMPACT was fabricated to test liquid patterning prior to injection molding. The prototype was designed using Solidworks (Dassault Systèmes) and fabricated using a 3D printer (Fig. 4, 3D SYSTEMS). After fabrication, the prototype was rinsed with isopropyl alcohol for 25 min and postcured with ultraviolet light at 385 nm for 30 min. To remove water and create a hydrophobic surface, the printed model was cured at 60 °C in an oven for 10 min. For biological experiments, poly(c-xylene) was deposited on the model by plasma-enhanced chemical vapor deposition (Lavida, Femtoscience). In addition, we tested the patterning conditions with four types of material: glass, 3M hydrophilic film, acrylic PET film, and PC film.

### Fabrication of the U-IMPACT

The polystyrene compartment was fabricated using an aluminum alloy mold core with machining and polishing. The injection clamping force was set to 130 tons, with a 15-s cycle time and a nozzle temperature of 220 °C. The polystyrene body was washed with isopropyl alcohol and deionized water for 10 min each and then treated with O_2_ plasma for 10 min. After processing, the chip was maintained in an oven at 60 °C for at least 7 days. We created a custom-designed double-sided adhesive film attached to a polystyrene body. Then, we attached a glass substrate (3 × 1 inches) to the other side of the adhesive film.

### Measurement of contact angle

The static and advancing contact angles were measured by the sessile drop method using a droplet analyzer (SmartDrop, Femtobiomed) with a droplet volume of 5 µL.

### Analytical model for patterning

The pressure of the liquid in the microchannels can be calculated based on surface energy changes^19^. At an infinitesimal volume d*V*, the change in the liquid–gas surface is d*A*_LG_, the solid–liquid surface is d*A*_SL_, and the surface tension is *γ*. Combining the total energy variation and Young’s equation, the pressure can be expressed as

$${\Delta}{{{P}}} = \gamma \left( {\frac{{{{{{\rm {d}}A}}}_{{{{\rm{LG}}}}}}}{{{{{\rm {{d}}V}}}}} - \cos \left( \theta \right)\frac{{{{{{\rm {d}}A}}}_{{{{\rm{SL}}}}}}}{{{{{\rm {{d}}V}}}}}} \right)$$

We analyzed two pressures, the advancing interface [${\Delta}{{{P}}}_{{{{\rm{forward}}}}}\left( {{\Delta}{{{P}}}_{{{\rm{f}}}}} \right)$] and bursting interface [${\Delta}{{{P}}}_{{{{\rm{burst}}}}}\left( {{\Delta}{{{P}}}_{{{\rm{b}}}}} \right)$]. To simplify the calculation, we neglected the free surface of the liquid interface, surface roughness of the channel, and round edge of the channel. The liquid interface can move forward only when the solid–liquid contact angle exceeds the critical advancing contact angle. In addition, the interface bulges until the contact angle with the new wall increases to the new advancing contact angle; the contact angle never exceeds 180°. Because the edge angle of the body is 90°, ∆*P*_b,1_ and ∆*P*_f,1_ can be expressed as

$${\Delta}{{{P}}}_{{{{\rm{b}}}},1} = \gamma \left( {\frac{2}{{{{{L}}}({{{t}}})}} - \frac{{{{{\rm{cos}}}}\theta _{{{\rm{b}}}}^ \ast + {{{\rm{cos}}}}\theta _{{{\rm{s}}}}^ \ast }}{{{{{h}}}_1}}} \right)$$

$${\Delta}{{{P}}}_{{{{\rm{f}}}},1} = \gamma \left( {\frac{2}{{{{{w}}}_1}} - \frac{{{{{\rm{cos}}}}\theta _{{{\rm{b}}}} + {{{\rm{cos}}}}\theta _{{{\rm{s}}}}}}{{{{{h}}}_1}}} \right)$$

$$\theta _{{{\rm{b}}}}^ \ast = {{{\rm{min}}}}\left( {\theta _{{{\rm{b}}}} + \frac{\pi }{2},\pi } \right),\,\theta _{{{\rm{s}}}}^ \ast = {{{\rm{min}}}}\left( {\theta _{{{\rm{s}}}},\pi } \right)$$

where the symbols represent the geometric parameters of the channel (*w*, width; *h*, height; *L*, length; *θ*_b_, advancing contact angle of body; *θ*_s_, advancing contact angle of substrate; *θ*_g_, advancing contact angle of fibrin gel; and ***γ***, surface tension). In addition, ∆*P*_b,2_ and ∆*P*_f,2_ can be expressed as

$${\Delta}{{{P}}}_{{{{\rm{b}}}},2} = \gamma \left( {\frac{2}{{{{{L}}}\left( {{{t}}} \right)}} - \frac{{{{{\rm{cos}}}}\theta _{{{\rm{b}}}}^ \ast + {{{\rm{cos}}}}\theta _{{{\rm{s}}}}^ \ast }}{{{{{h}}}_2}}} \right)$$

$${\Delta}{{{P}}}_{{{{\rm{f}}}},2} = \gamma \left( {\frac{{1 - {{{\rm{cos}}}}\theta _{{{\rm{b}}}}}}{{{{{w}}}_2}} - \frac{{{{{\rm{cos}}}}\theta _{{{\rm{b}}}} + {{{\rm{cos}}}}\theta _{{{\rm{s}}}}}}{{{{{h}}}_2}} + \frac{{{{{h}}}_1\left( {{{{\rm{cos}}}}\theta _{{{\rm{b}}}} - {{{\rm{cos}}}}\theta _{{{\rm{g}}}}} \right)}}{{{{{w}}}_2{{{h}}}_2}}} \right)$$

$$\theta _{{{\rm{b}}}}^ \ast = {{{\rm{min}}}}\left( {\theta _{{{\rm{b}}}} + \frac{\pi }{2},\pi } \right),\,\theta _{{{\rm{s}}}}^ \ast = {{{\rm{min}}}}\left( {\theta _{{{\rm{s}}}},\pi } \right)$$

We established the design rules based on these equations and plotted the graph with MATLAB (MathWorks) under the assumption of water at 25 °C. The pressure difference (${\Delta}{{{P}}}_{{{{\rm{b}}}},1} - {\Delta}{{{P}}}_{{{{\rm{f}}}},1}$) and ∆*P*_f,1_ were plotted against height or width change, with the width fixed at *w*_1_ = 2 mm or the height at *h*_1_ = 0.25 mm, respectively. When *h*_1_ = 0.25 mm, the pressure difference (${\Delta}{{{P}}}_{{{{\rm{b}}}},2} - {\Delta}{{{P}}}_{{{{\rm{f}}}},2}$) and ${\Delta}{{{P}}}_{{{{\rm{f}}}},2}$ were plotted against the height or width change with the width fixed at ${{{w}}}_2 = 1\,{{{\rm{mm}}}}$ or the height at ${{{h}}}_2 = 0.45\,{{{\rm{mm}}}}$, respectively.

### Simulation analysis

We utilized COMSOL Multiphysics software to perform simulations of concentrations and concentration gradients using the U-IMPACT. For finite element analysis of the biochemical diffusion effect, a computer-aided design of the platform was imported, and the transport of diluted species with porous properties was added to the U-IMPACT. Although the flow could be calculated from the height difference of the medium change, the height difference was equilibrated rapidly. Therefore, we estimated the diffusion term alone; we did not determine the convection term.

### Cell culture

All procedures and experiments were approved by the Institutional Animal Care and Use Committee of Seoul National University (SNU-201016-1-1). All cells were cultured in a 5% CO_2_ incubator at 37 °C. We used human umbilical vein endothelial cells (HUVECs, Lonza) at passage 4 and normal human LFs (Lonza) at passage 5 to reconstruct vessel networks in the chip^10,30^. HUVECs and LFs were cultured in endothelial growth medium-2 (PromoCell) and fibroblast growth medium-2 (Lonza), respectively. Human glioblastoma cells (U87MG, Korean Cell Line Bank) were used for tumor spheroid formation, and the cells were cultured in Dulbecco’s modified Eagle’s medium (Sigma) supplemented with 10% FBS and 1% penicillin–streptomycin.

### Spheroid preparation

Tumor spheroids were formed using U87MG, HCT116, and SW480 cells combined with LFs^10^. The tumor cell and LF suspensions were mixed at a 1:1 ratio (5000 total cells per well) with a 1% volume ratio to hydrogel (Matrigel, Corning). Spheroids were grown in 96-well plates with U-shaped wells (Sumitomo Bakelite) for 2 days. Each spheroid was introduced into the chip with the HUVEC and LF suspension, along with fibrin gel, to reconstruct a perfusable vessel network with a tumor spheroid. Neurosphere preparation was performed in a nearly identical manner^32^. Briefly, iNSCs were dissociated with Accutase (Gibco) to generate single-cell suspensions. The iNSC suspension was plated in each well of a 96-well plate (Corning) at 9000 cells per well. The neurospheres were incubated for 3 days in NSC maintenance medium as described previously.

### Immunocytochemistry

The samples were fixed with 4% paraformaldehyde (Thermo) for 20 min at room temperature and then treated with 0.2% Triton X-100 for 15 min. Each sample was then mixed with 4% bovine serum albumin (Millipore) and incubated at 4 °C overnight. Endothelial cells and tumor cells were marked using 488 Ulex europaeus agglutinin 1 (Vector Laboratories), green fluorescent protein-tagged cells (U87MG), and Alexa Fluor 594‐tagged variants of anti‐epithelial cell adhesion molecule (BioLegend) for 2 days. Nuclei were stained with DAPI (Life Technologies) for 15 min. Neural cells were stained with Tuj1 (1:200) and Oligo2 (1:200). All images were acquired using a confocal microscope (Nikon Ti 2) equipped with lasers (excitation wavelengths: 405, 488, and 594 nm).

## Supplementary information

Supplemental Material

Supplementary Information

Supplementary Figures

Figure S1

Figure S2

Figure S3

Figure S4

## References

[1] Huh D (2010). Reconstituting organ-level lung functions on a chip. Science.

[2] Bang S, Jeong S, Choi N, Kim HN (2019). Brain-on-a-chip: a history of development and future perspective. Biomicrofluidics.

[3] Moradi, E., Jalili-Firoozinezhad, S. & Solati-Hashjin, M. Microfluidic organ-on-a-chip models of human liver tissue. *Acta Biomater*. **116**, 67–83 (2020). 10.1016/j.actbio.2020.08.041 32890749

[4] Esch, E. W., Bahinski, A. & Huh, D. Organs-on-chips at the frontiers of drug discovery. *Nat. Rev. Cancer* **14**, 4 (2015). 10.1038/nrd4539 PMC4826389 25792263

[5] Huh DD (2017). Polysaccharide-based nanoparticles for gene delivery. FASEB J..

[6] Dewhirst MW, Secomb TW (2017). Transport of drugs from blood vessels to tumour tissue. Nat. Rev. Cancer.

[7] Whiteside T (2008). The tumor microenvironment and its role in promoting tumor growth. Oncogene.

[8] Joyce JA (2005). Therapeutic targeting of the tumor microenvironment. Cancer Cell.

[9] Shirure VS (2018). Tumor-on-a-chip platform to investigate progression and drug sensitivity in cell lines and patient-derived organoids. Lab Chip.

[10] Ko J (2019). Tumor spheroid-on-a-chip: a standardized microfluidic culture platform for investigating tumor angiogenesis. Lab Chip.

[11] Xiao Y (2019). The impact of preoperative fibrinogen-albumin ratio on mortality in patients with acute ST-segment elevation myocardial infarction undergoing primary percutaneous coronary intervention. Adv. Sci..

[12] Probst, C., Schneider, S. & Loskill, P. High-throughput organ-on-a-chip systems: Current status and remaining challenges. *Curr. Opin. Biomed. Eng*. **6**, 33–41 (2018)

[13] Van Meer, B. et al. Small molecule absorption by PDMS in the context of drug response bioassays. *Biochem. Biophys. Res. Commun.* **482**, 323–328 (2017). 10.1016/j.bbrc.2016.11.062 PMC5240851 27856254

[14] Mukhopadhyay R (2007). AFM tells elements apart. Anal. Chem..

[15] Lee Y (2018). On-chip oocyte denudation from cumulus–oocyte complexes for assisted reproductive therapy. Lab Chip.

[16] Van Duinen, V., Trietsch, S. J., Joore, J., Vulto, P. & Hankemeier, T. Microfluidic 3D cell culture: from tools to tissue models. *Curr. Opin. Biotechnol*. **35**, 118–126 (2015) 10.1016/j.copbio.2015.05.002 26094109

[17] Van Duinen V (2017). 96 perfusable blood vessels to study vascular permeability in vitro. Sci. Rep..

[18] Berthier E, Young EW, Beebe D (2012). Engineers are from PDMS-land, Biologists are from Polystyrenia. Lab Chip.

[19] Casavant, B. P. et al. Suspended microfluidics. *Proc. Natl Acad. Sci. USA* **110**, 25 (2013). 10.1073/pnas.1302566110 PMC3690848 23729815

[20] Vulto P (2011). Phaseguides: a paradigm shift in microfluidic priming and emptying. Lab Chip.

[21] Yu J (2019). Reconfigurable open microfluidics for studying the spatiotemporal dynamics of paracrine signalling. Nat. Biomed. Eng..

[22] Yi H-G, Lee H, Cho D-W (2017). 3D printing of organs-on-chips. Bioengineering.

[23] Lee S-R (2019). Enhanced bioactivity of titanium-coated polyetheretherketone implants created by a high-temperature 3D printing process. Biofabrication.

[24] Occhiello E, Morra M, Cinquina P, Garbassi F (1992). Hydrophobic recovery of oxygen-plasma-treated polystyrene. Polymer.

[25] Huang, C. P. et al. Engineering microscale cellular niches for three-dimensional multicellular co-cultures. *Lab Chip* **9**, 12 (2009). 10.1039/b818401a PMC3758562 19495458

[26] Kim, S., Lee, H., Chung, M. & Jeon, N. L. Engineering of functional, perfusable 3D microvascular networks on a chip. *Lab Chip* **13**, 8 (2013). 10.1039/c3lc41320a 23440068

[27] Kim S, Chung M, Ahn J, Lee S, Jeon NL (2016). Dual-patterned immunofiltration (DIF) device for the rapid efficient negative selection of heterogeneous circulating tumor cells. Lab Chip.

[28] Azizgolshani H (2021). High-throughput organ-on-chip platform with integrated programmable fluid flow and real-time sensing for complex tissue models in drug development workflows. Lab Chip.

[29] Lee S (2021). Extracellular cAMP: the past and visiting the future in cAMP‐enriched extracellular vesicles. Adv. Biol..

[30] Kim S (2021). Mechanobiological conceptual framework for assessing stem cell bioprocess effectiveness. Biotechnol. Bioeng..

[31] Jokinen V, Suvanto P, Franssila S (2012). Oxygen and nitrogen plasma hydrophilization and hydrophobic recovery of polymers. Biomicrofluidics.

[32] Yu K-R (2015). Rapid and efficient direct conversion of human adult somatic cells into neural stem cells by HMGA2/let-7b. Cell Rep..

