# Supplementary material for: U-IMPACT: a universal 3D microfluidic cell culture platform
Source: Microsyst Nanoeng. 2022 Dec 5;8:126. doi: 10.1038/s41378-022-00431-w (PMC9719897; doi:10.1038/s41378-022-00431-w)
Supplement: Supplementary file 2 — Supplementary Information [file 41378_2022_431_MOESM2_ESM.docx]

**U-IMPACT: A Universal 3D Microfluidic Cell Culture Platform**

Seung-Ryeol Lee^1^**^†^**, Youngtaek Kim^1^**^†^**, Suryong Kim^1^**^†^**, Jiho Kim^1^, Seonghyuk Park^1^, Stephen Rhee^1^, Dohyun Park^1^, Byungjun Lee^2^, Kyusuk Baek^2^, Ho-Young Kim^1,3^ and Noo Li Jeon^1,3,4*^

^1^Department of Mechanical Engineering, Seoul National University, Seoul, Republic of Korea

^2^Qureator Incorporation, Seoul, Republic of Korea

^3^Institute of Advanced Machines and Design Seoul National University, Seoul, Republic of Korea

^4^Institute of Bioengineering, Seoul National University, Seoul, Republic of Korea

†These authors contributed equally to this work

*****Corresponding Author: **Noo Li Jeon, Ph.D.**

E-Mail: njeon@snu.ac.kr

Supplementary Movie 1. Real-time monitoring of 2-μm microbead (red) movement in perfusable vascular network (green). Scale bar, 50 μm.
