# Supplementary material for: U-IMPACT: a universal 3D microfluidic cell culture platform
Source: Microsyst Nanoeng. 2022 Dec 5;8:126. doi: 10.1038/s41378-022-00431-w (PMC9719897; doi:10.1038/s41378-022-00431-w)
Supplement: Supplementary file 3 — Supplementary Figures [file 41378_2022_431_MOESM3_ESM.docx]

Supplementary Figure 4. Simulation of the growth factor concentration and concentration gradient. Simulation of growth factor concentration (a) and concentration gradient (b) in the PDMS-based platform developed previously by our group. (c) Distribution of growth factor concentration where ECs were seeded (B-B′). (d) Simulation of the concentration gradient in the angiogenesis model. (e) Distribution of the concentration gradient in the angiogenesis model (B-B′). (f) Simulation of the concentration gradient in the vascularized tumor model. (g) Distribution of the concentration gradient in the vascularized tumor model (B-B′).

Supplementary Figure 2. Experimental patterning results on the MC. (a) Experimental results with a body contact angle of 45° using the 3DP prototype. The liquid tended to invade other regions in accordance with SCF. (b) Graph of the pressure difference ($\boldsymbol{\Delta}\boldsymbol{P}_{\boldsymbol{b,1}}\boldsymbol{-\Delta}\boldsymbol{P}_{\boldsymbol{f,1}}$) with a height of 0.25 mm. (c) Graph of $\boldsymbol{\Delta}\boldsymbol{P}_{\boldsymbol{f}}$ with a height of 0.25 mm and body contact angle of 70°. Graph of $\boldsymbol{\Delta}\boldsymbol{P}_{\boldsymbol{f}}$ with a width of 2 mm (d) or height of 0.25 mm (e) and body contact angle of 94°. Based on the parameter analysis results, we experimentally set $\boldsymbol{\Delta}\boldsymbol{P}_{\boldsymbol{f}}$ to −200 Pa as the maximum threshold for robust first patterning (green circles, success; red triangles, failure).

Supplementary Figure 3. Experimental patterning results on the side channels. (a) Graph of the pressure difference ($\boldsymbol{\Delta}\boldsymbol{P}_{\boldsymbol{b,2}}\boldsymbol{-\Delta}\boldsymbol{P}_{\boldsymbol{f,2}}$) at a 0.45 mm height. (b) Graph of $\boldsymbol{\Delta}\boldsymbol{P}_{\boldsymbol{f}}$ at a 0.45 mm height and 70° of body contact angle. Graph of $\boldsymbol{\Delta}\boldsymbol{P}_{\boldsymbol{f}}$ at 1 mm width (c) or 0.45 mm height (d) and 94° of body contact angle. Based on the parameter study, we experimentally set $\boldsymbol{\Delta}\boldsymbol{P}_{\boldsymbol{f}}$ to -50 Pa as the maximum threshold for robust first patterning (green circle; success, red triangle; failure).

Supplementary Figure 1. Static and advancing contact angle of materials. (a) Static contact angles of the body and substrate. We measured material properties using the sessile drop method with 5-µL droplets of deionized water. (b) Advancing contact angles of the body and substrate. Advancing contact angles were slightly greater than static contact angles. Hydrophobic recovery of polystyrene (c) and 3DP (d) after plasma treatment. The contact angle recovered almost to its original value over 1 month.
