# Supplementary material for: U-IMPACT: a universal 3D microfluidic cell culture platform
Source: Microsyst Nanoeng. 2022 Dec 5;8:126. doi: 10.1038/s41378-022-00431-w (PMC9719897; doi:10.1038/s41378-022-00431-w)

Supple. Fig 1.

a

	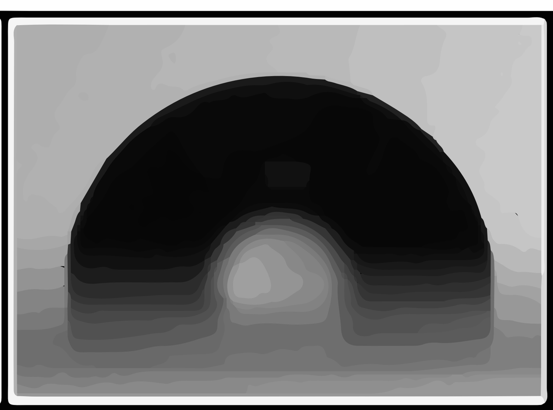	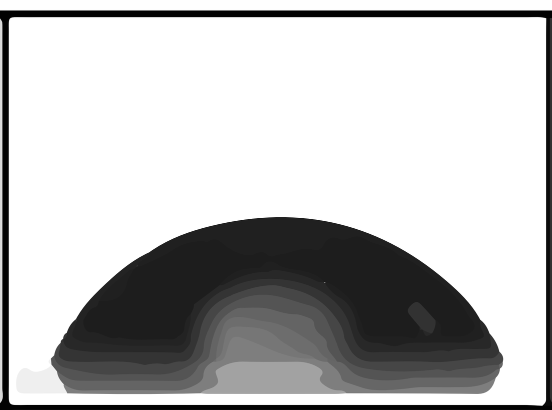	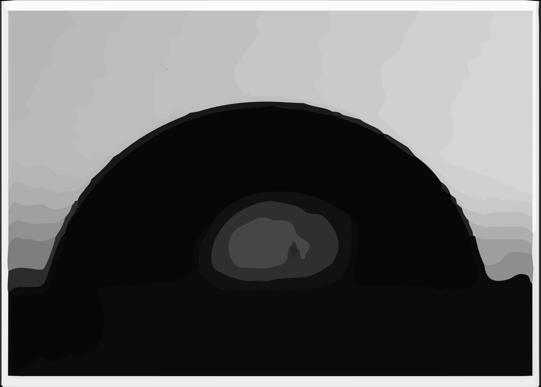	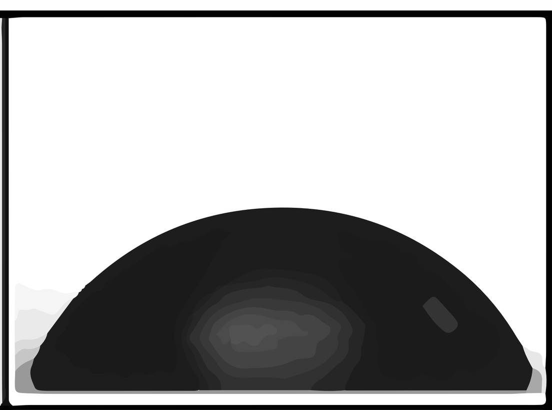	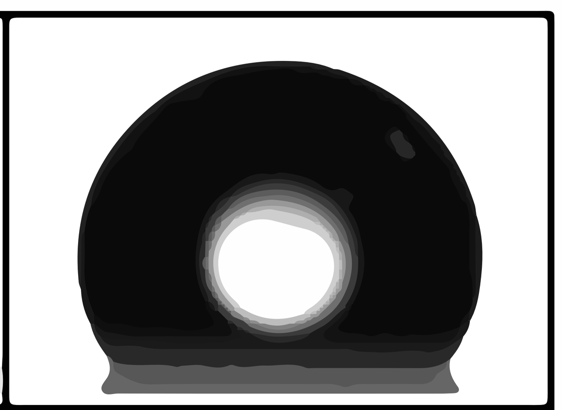
**Body material**	PS	PS w/ cleaning	3D printer	3DP w/ cleaning	Fibrin gel
**Contact angle(˚)**	96.3	66.9 (day 15)	83.0	68.9 (day 15)	127.3
	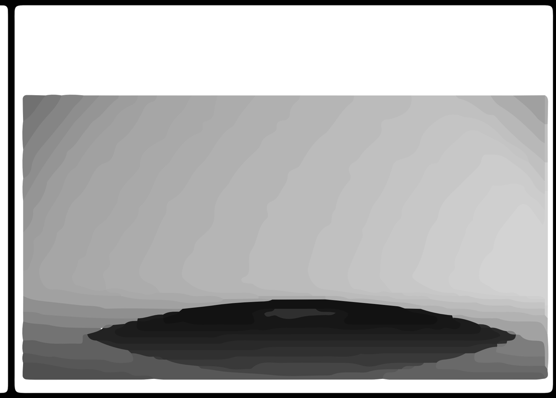	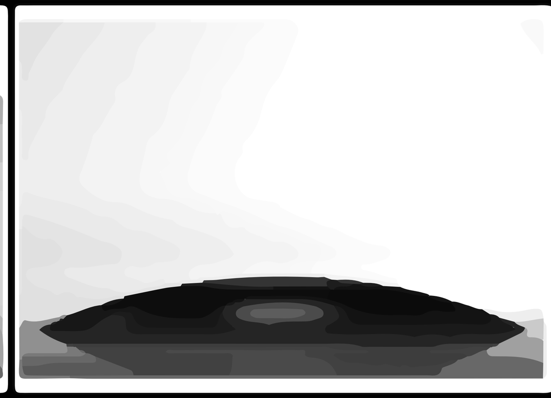	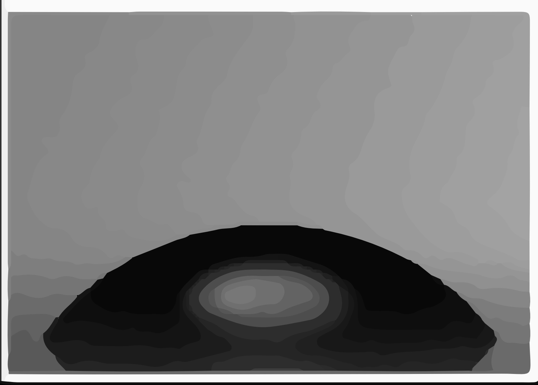	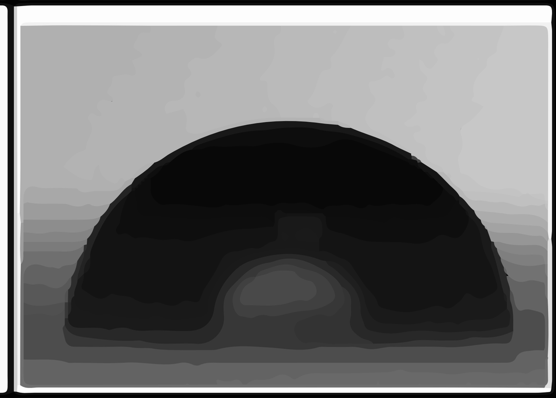	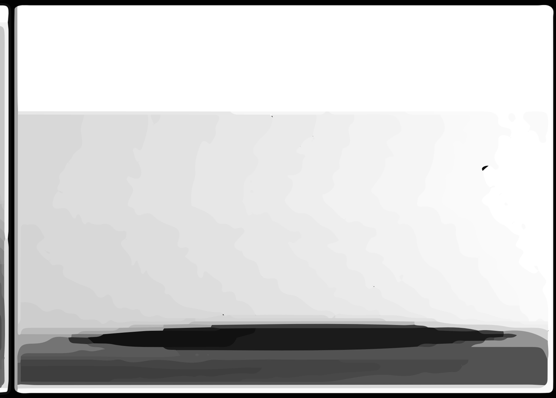
**Substrate Material**	Glass	3M hydrophilic film	Acrylic PET film	PC film	PC film after plasma
**Contact angle(˚)**	19.5	26.9	53.6	91.3	~ 0

b

	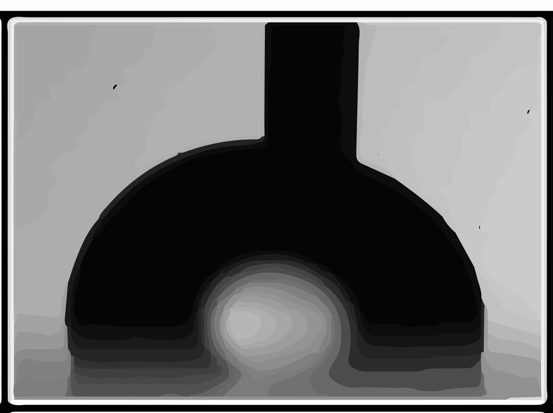	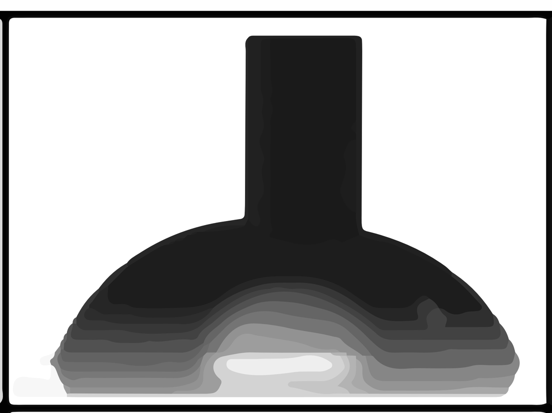	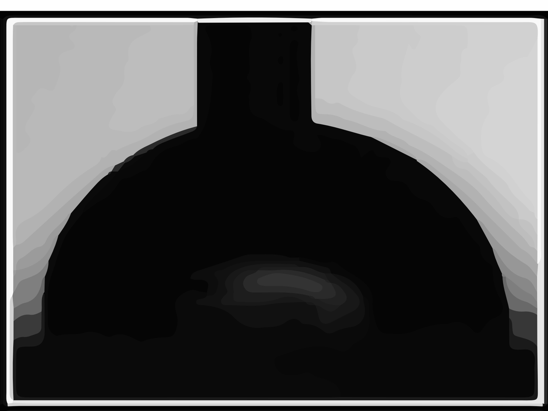	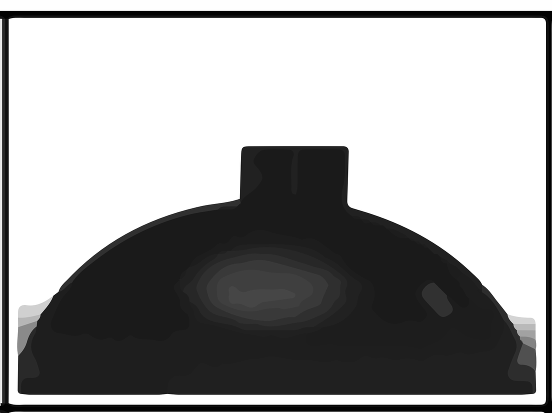	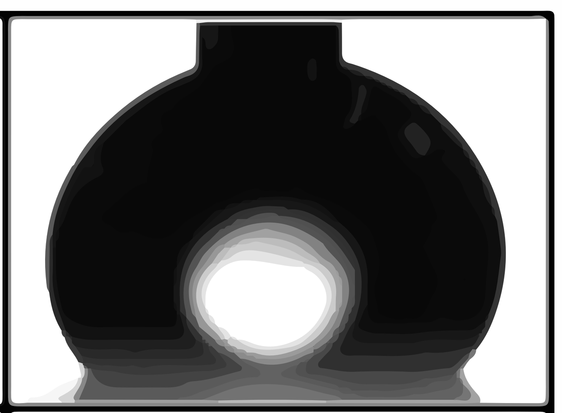
**Body material**	PS	PS w/ cleaning	3D printer	3DP w/ cleaning	Fibrin gel
**Advancing contact angle(˚)**	106.7	70.9 (day 15)	94.0	69.5 (day 15)	135.2
	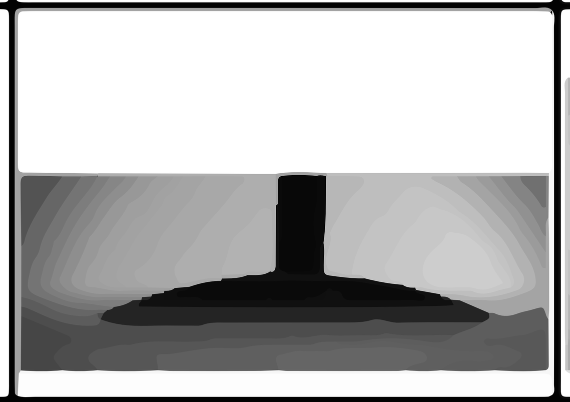	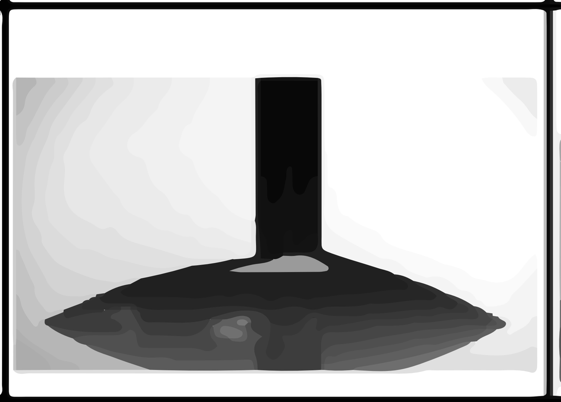	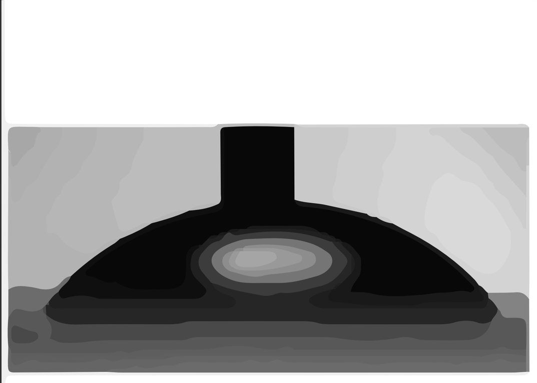	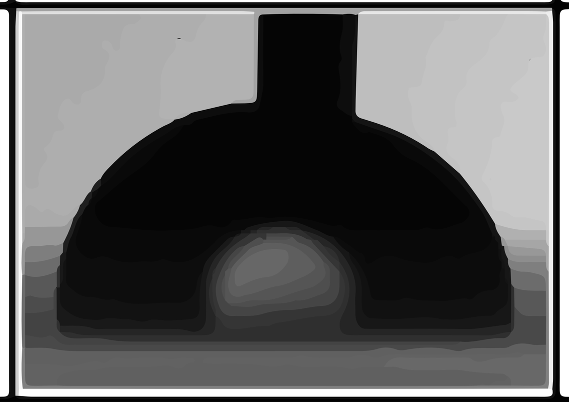	-
**Substrate Material**	Glass	3M hydrophilic film	Acrylic PET film	PC film	PC film after plasma
**Advancing contact angle(˚)**	22.3	30.8	55.3	97.2	-

c

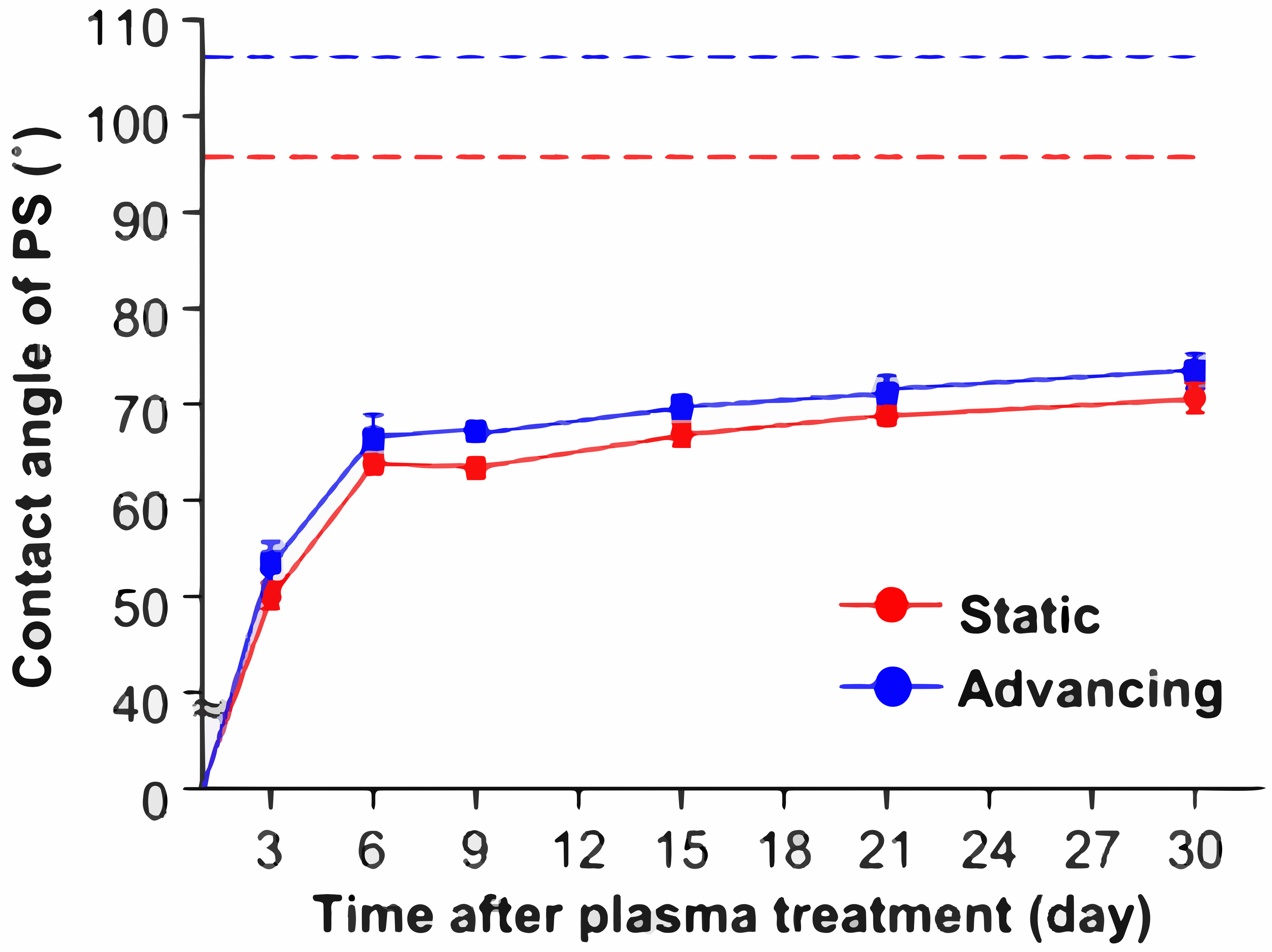

d

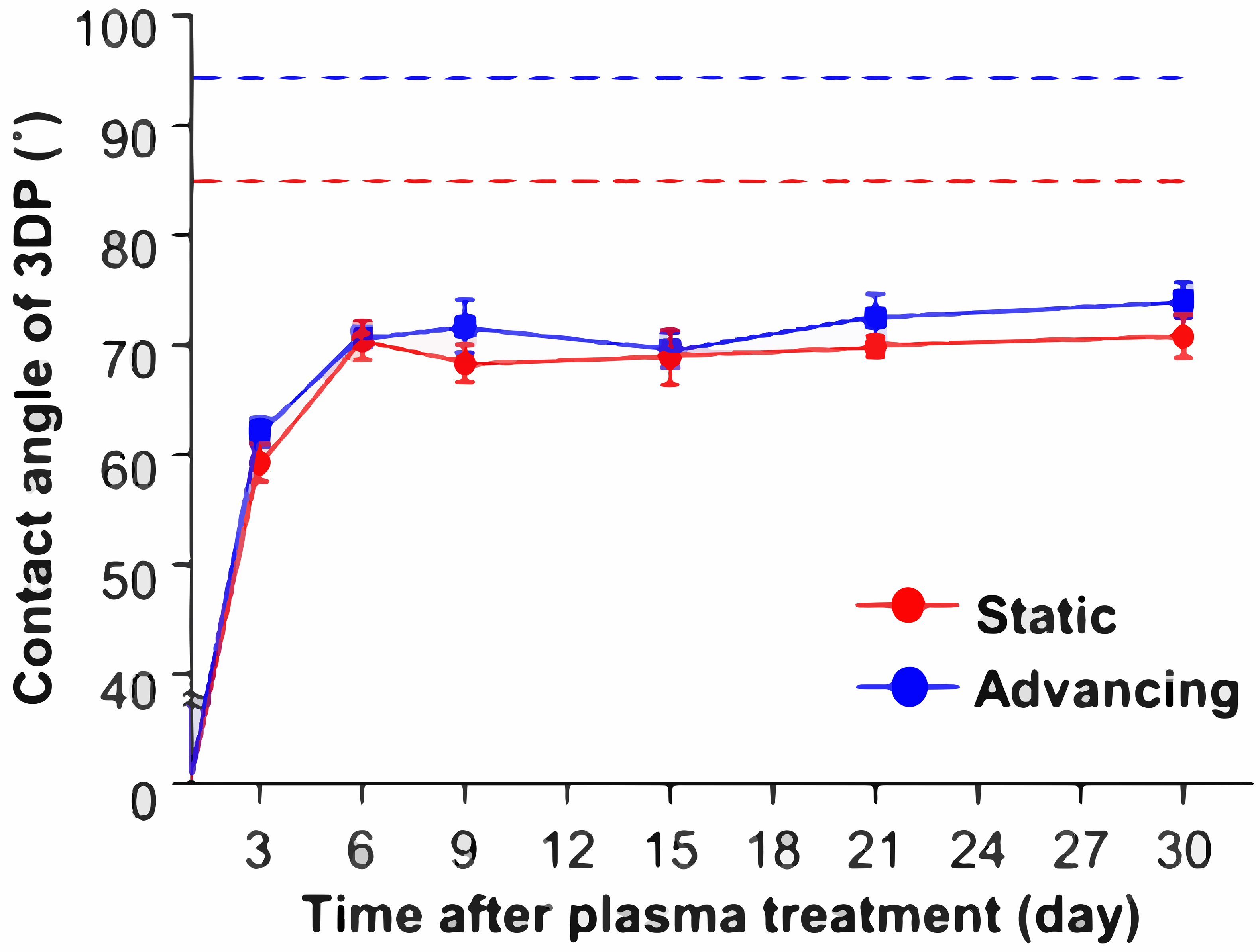

Supplement: Supplementary file 4 — Figure S1 [file 41378_2022_431_MOESM4_ESM.pdf]
