# Supplementary material for: U-IMPACT: a universal 3D microfluidic cell culture platform
Source: Microsyst Nanoeng. 2022 Dec 5;8:126. doi: 10.1038/s41378-022-00431-w (PMC9719897; doi:10.1038/s41378-022-00431-w)
Supplement: Supplementary file 5 — Figure S2 [file 41378_2022_431_MOESM5_ESM.pdf]

Supple. Fig 2.

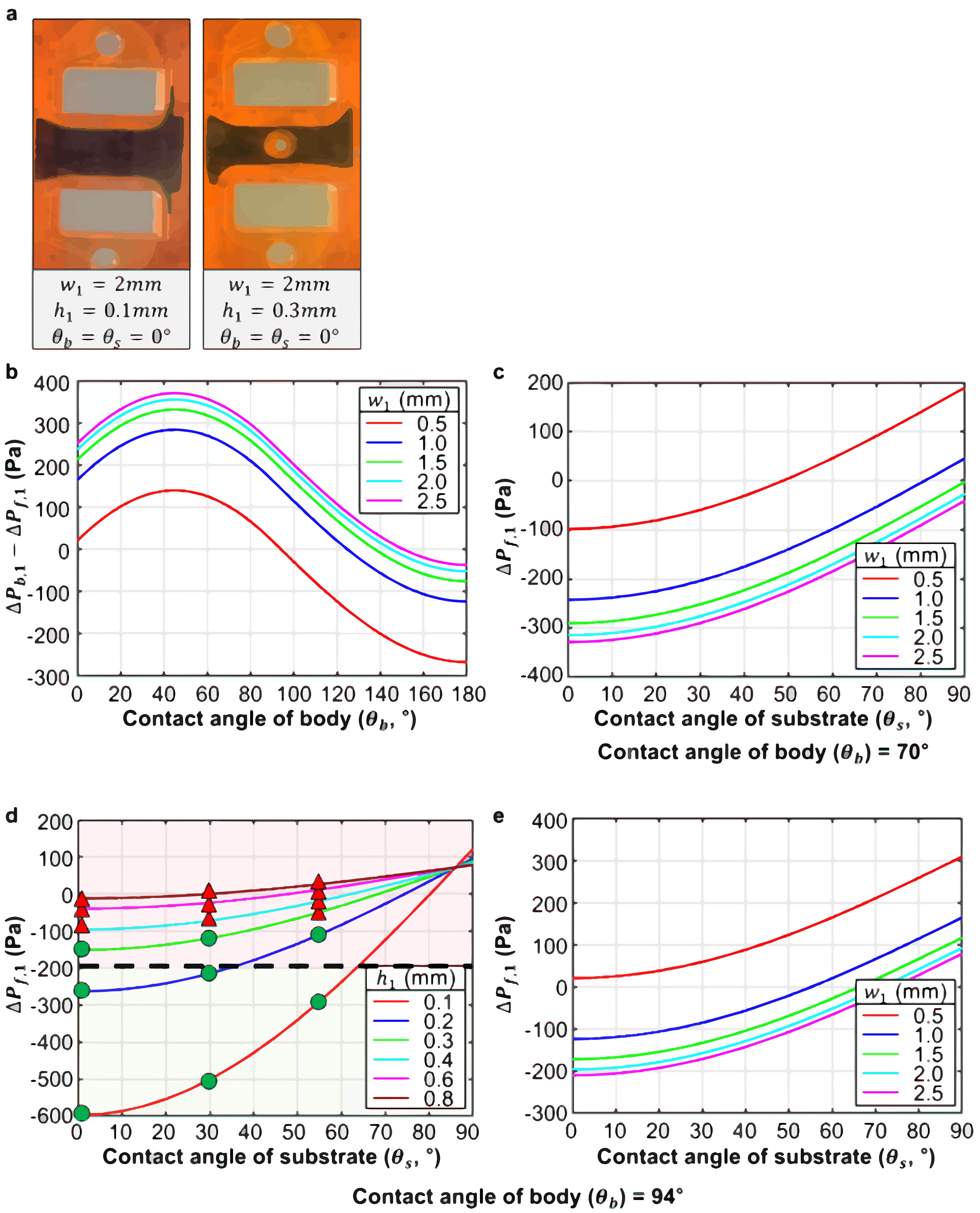
a
w1 = 2mm
h1 = 0.1mm
θb = θs = 0°
w1 = 2mm
h1 = 0.3mm
θb = θs = 0°
b
ΔPb,1 − ΔPf,1 (Pa)
Contact angle of body (θb, °)
w1 (mm)
0.5
1.0
1.5
2.0
2.5
c
ΔPf,1 (Pa)
Contact angle of substrate (θs, °)
Contact angle of body (θb) = 70°
d
ΔPf,1 (Pa)
h1 (mm)
0.1
0.2
0.3
0.4
0.6
0.8
Contact angle of substrate (θs, °)
e
ΔPf,1 (Pa)
Contact angle of substrate (θs, °)
Contact angle of body (θb) = 94°
