# Supplementary material for: U-IMPACT: a universal 3D microfluidic cell culture platform
Source: Microsyst Nanoeng. 2022 Dec 5;8:126. doi: 10.1038/s41378-022-00431-w (PMC9719897; doi:10.1038/s41378-022-00431-w)
Supplement: Supplementary file 6 — Figure S3 [file 41378_2022_431_MOESM6_ESM.pdf]

Supple. Fig 3.

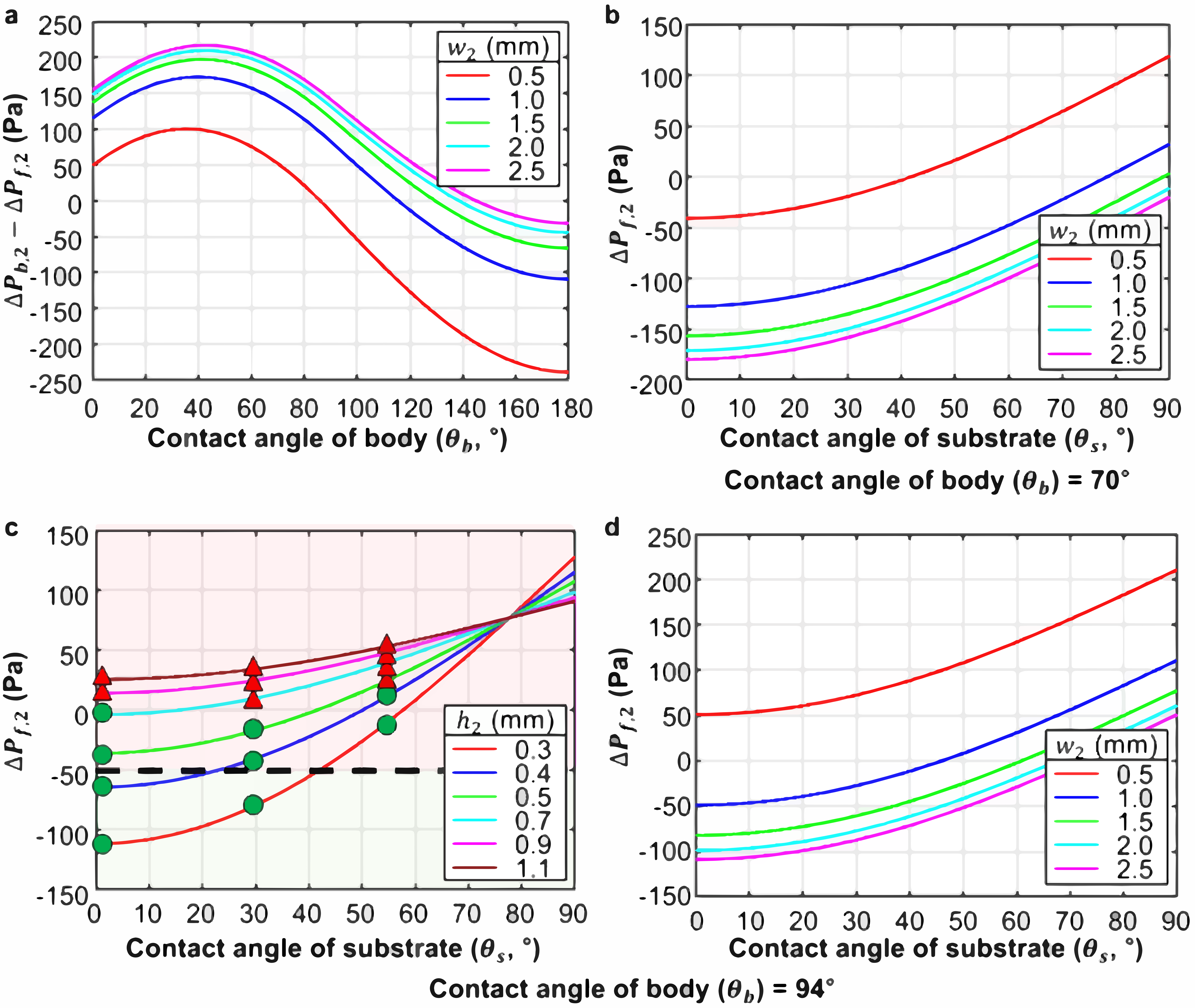
a
b
c
d
$\Delta P_{b,2} - \Delta P_{f,2}$ (Pa)
Contact angle of body (θ_b, °)
w_2 (mm)
0.5
1.0
1.5
2.0
2.5
$\Delta P_{f,2}$ (Pa)
Contact angle of substrate (θ_s, °)
Contact angle of body (θ_b) = 70°
h_2 (mm)
0.3
0.4
0.5
0.7
0.9
1.1
Contact angle of body (θ_b) = 94°
