# Supplementary material for: U-IMPACT: a universal 3D microfluidic cell culture platform
Source: Microsyst Nanoeng. 2022 Dec 5;8:126. doi: 10.1038/s41378-022-00431-w (PMC9719897; doi:10.1038/s41378-022-00431-w)
Supplement: Supplementary file 7 — Figure S4 [file 41378_2022_431_MOESM7_ESM.pdf]

Supple. Fig 4.

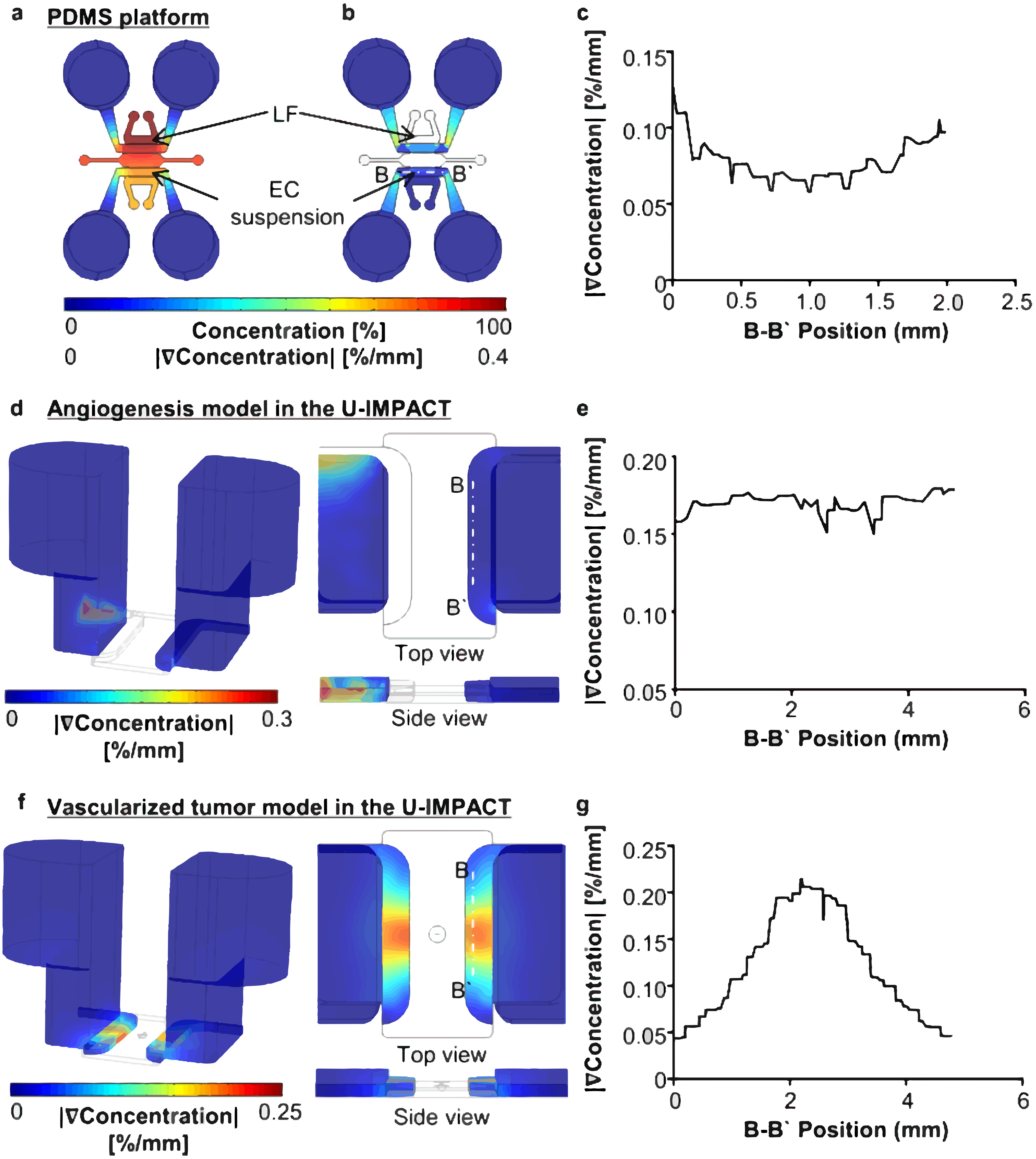

a
PDMS platform
LF
EC
suspension
b
B
B`
0
Concentration [%]
100
0
|∇Concentration| [%/mm]
0.4
c
|∇Concentration| [%/mm]
0.15
0.10
0.05
0
0
0.5
1.0
1.5
2.0
2.5
B-B` Position (mm)
d
Angiogenesis model in the U-IMPACT
B
B`
Top view
Side view
0
|∇Concentration| [%/mm]
0.3
e
|∇Concentration| [%/mm]
0.20
0.15
0.10
0.05
0
2
4
6
B-B` Position (mm)
f
Vascularized tumor model in the U-IMPACT
B
B`
Top view
Side view
0
|∇Concentration| [%/mm]
0.25
g
|∇Concentration| [%/mm]
0.25
0.20
0.15
0.10
0.05
0
0
2
4
6
B-B` Position (mm)
